# Orbital-based bonding analysis in solids

**DOI:** 10.1039/d5sc02936h

**Published:** 2025-06-09

**Authors:** Peter C. Müller, Linda S. Reitz, David Hemker, Richard Dronskowski

**Affiliations:** a Chair of Solid-State and Quantum Chemistry, Institute of Inorganic Chemistry, RWTH Aachen University D-52056 Aachen Germany drons@HAL9000.ac.rwth-aachen.de +49-241 80 92642 +49-241 80 93642

## Abstract

As of today, there is certainly no doubt about the quantum character of the atomistic world, most straightforwardly calculated by using wave mechanics and Schrödinger's fundamental equation from 1926. Even though one century has passed, the paramount importance of the wave function, which determines everything down to the last detail, remains unchanged, and the wave function is most conveniently approximated by a combination of orbitals, one-electron wave functions for atoms, molecules, and also solids. And it is precisely this “orbital basis” that serves as a gateway to understanding the very interactions that cause atoms to condense into solids, just like for molecules. The analysis of quantum-chemical interactions and the nature of the chemical bonding between atoms in solids by use of orbitals will be our topic in this perspective, starting with the glorious past, going over to the current practice and, of course, the magnificent prospects for the future. As electronic structures for periodic solids are most often calculated using plane waves (instead of orbitals), for simple reasons of translational symmetry and Bloch's fundamental theorem, a unitary transformation to atomic or molecular orbitals is needed for final inspection, technically solved by the LOBSTER quantum-chemistry package. LOBSTER allows for the calculation of wave function-based atomic charges, various population analyses and periodic bonding indicators, first-principles bond orders, two- and multi-centre bonding analysis, fragment-molecular analysis, and a lot more. All those techniques are illustrated from three solid-state systems deriving from carbonate chemistry.

## Abstract 2

The wave function remains central to understanding chemical bonding, with orbitals allowing to analyse various interactions most conveniently, as exemplified for molecular solids using the LOBSTER package extracting them from plane-wave calculations.

## Graphical abstract

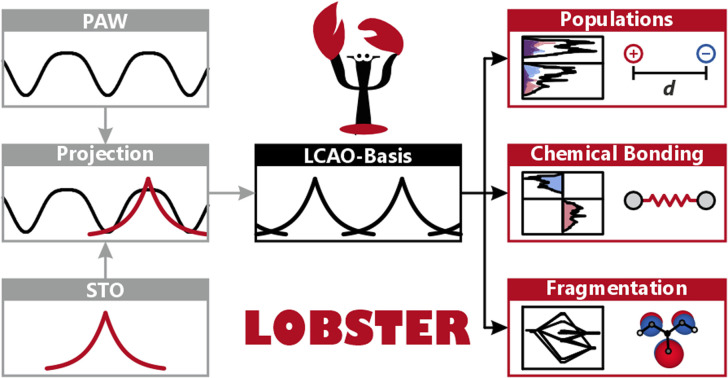

## 1 Introduction

The concept of the “chemical bond” is probably the most important ingredient in the intellectual toolbox of any chemist. While the term itself was first coined by the English chemist Frankland in 1866 already,^1^ its current notion was essentially shaped by Lewis in 1916,^2^ even before the advent of Heisenberg's quantum mechanics in 1925,^3^ this being the reason why Lewis had difficulties understanding how two negatively charged electrons would approach each other in a “single” bond represented by a simple dash symbol, *e.g.*, H–H. Already then, however, Lewis recognised the importance of distinguishing ionic and covalent bonding in chemical systems, and chemists quickly picked up on the idea and developed the model into the incredibly successful concept it is today. The discovery of quantum mechanics in the form of wave mechanics, that is, in the formulation of Schrödinger's famous equation from 1926 (ref. 4 and 5) quickly led chemistry-affine physicists, such as Heitler and London, to search for the origins of the chemical bond in the emerging quantum theory. While working as postdocs with Schrödinger, Heitler and London discovered the chemical bond's nature in the first quantum-chemical solution to the H_2_ wave function, namely by the so-called valence-bond (VB) approach assuming both electrons being strictly “correlated”, that is, without ionic terms.^6^ This topic is large enough to be discussed on its own, so we refer the interested reader to a recent review.^7^ In particular, Heitler and London recognised the importance of interfering wave functions, at that time dubbed “Schwebungsphänomen” in the German original. (Atomic) wave functions interfere, that is what covalent bonding between atoms is all about, no matter how it is actually calculated.

This first bonding analysis on quantum-chemical footing is the fundament of a rich set of theories, tools, and algorithms that would be developed in the following century, until today.^8^ We will shortly review the historic developments of chemical bonding analyses within this section, followed by a brief overview on LOBSTER,^9–11^ a software suite implementing every major development in the field of wave function-based bonding analysis within a periodic solid-state context. This is followed by several sections detailing the mathematical backgrounds, results, and interpretational strength of the bonding analyses discussed in this article.

**Fig. 1:**
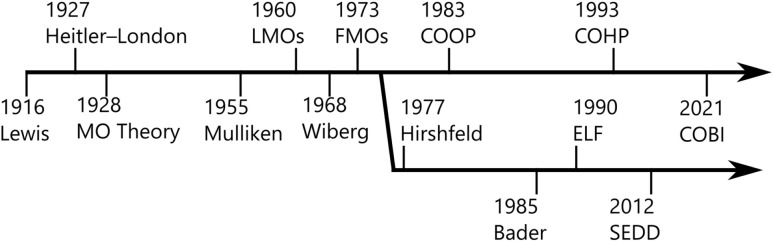
A brief history of key developments in quantum chemistry alluding to chemical bonding.

Fig. 1 summarises the history of what we think are key developments in the context of quantum chemistry-based tools targeted at bonding analyses or at least alluding to chemical bonding. Shortly after the proof of concept was delivered by Heitler and London, Condon as well as Mulliken and Hund found yet another and quite different solution to the same problem, the molecular-orbital theory (MO theory),^12–16^ namely by assuming the electrons being “uncorrelated”, totally independent from each other, thereby also allowing for “ionic” terms, in contrast to VB theory. By doing so, they offered an efficient ansatz to approach electronic wave functions of complex molecular systems based on a superposition of overlapping atomic orbitals—an approach that inspired and nowadays goes under the name linear combination of atomic orbitals (LCAO). The rather delocalised nature of the solutions to the MO wave functions led to hesitation within the chemical community at first since models based on valence-bond theory, in particular those invented by Pauling^17^ looked a little more intuitive or “chemical”, but success stories using Hückel MO theory on aromatic molecules,^18^ the Woodward–Hoffmann^19^ rules based on the shapes of molecular orbitals and also Fukui functions^20^ targeting molecular reactivities quickly led to a broad acceptance of MO theory among chemists.

Still pursuing a detailed understanding of chemical bonding, chemists started developing tools to divide and conquer the delocalised molecular wave function into quantities that are suitable for analyzing the chemical bond between two (or even more) atomic centres. First was Mulliken who suggested an electronic population analysis, yielding overlap populations and atomic charges, allowing to investigate covalent and ionic bonding between two atoms in an MO-like wave function.^21–24^ An alternative to Mulliken's population analysis can be achieved when using an orthonormal basis set, *e.g.*, by orthogonalising the atomic-orbital basis first using Löwdin's symmetric orthogonalization.^25^ Foster and Boys,^26^ Edmiston and Ruedenberg,^27^ and Pipek and Mezey^28^ came up with protocols to generate so-called localised molecular orbitals, the first developments starting in 1960. Using the fact that, within MO theory approaches, the overall wave function is invariant regarding unitary transformations among the molecular orbitals, one may generate a set of maximally localised molecular orbitals that perfectly reflect early ideas of single, double or triple bonds as formulated by Lewis, even including “hybridised” (that is, mixed) atomic orbitals. The localisation idea was later introduced to the solid state by our physicist friends *via* so-called maximally localised Wannier functions (MLWF),^29^ albeit the protocols to generate these MLWFs are more involved.

This rather qualitative analysis of quantum-mechanical bond orders was later supplemented by Wiberg's quantitative bond index,^30^ which analyses the density matrix (incorporating the eigenvectors), obtained from the molecular orbital LCAO coefficients, to determine bond orders. The idea was further generalised by Mayer^31–33^ to non-orthogonal orbital bases. The year 1973 saw yet another profoundly chemical idea, namely an approach to investigate bonding between fragments (each comprised of several atoms), rather than between atoms, in order to further cater the chemist's view of matter, where molecular entities would show distinct properties, different from the individual atoms they are composed of. Hoffmann and coworkers developed the fragment molecular orbital (FMO) method to obtain molecular orbitals strictly localised on a fragment within a larger chemical compound, highly useful when trying to investigate interactions between different fragments (think about functional groups) that together form a whatever complex compound.^34^ In 1983, Hughbanks and Hoffmann generalised the Mulliken overlap population to periodic wave functions by means of the Crystal Orbital Overlap Population (COOP), offering a quantum-chemical bonding analysis for solid-state chemists for the first time.^35^1
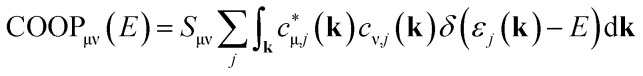


Without doubt, COOP completely changed the way solid-state chemists thought about solids because up to 1983 essentially every periodic solid was oversimplified as being ionic, whether truly ionic (such as NaCl) or not. Even far more covalent materials such as GaP were often written (and “understood”) as Ga^3+^P^3−^ before the advent of COOP.

In the 1960s, when density-functional theory (DFT) originating from metal physics^36–39^ slowly gained attraction in the solid-state field and, much later, also in the molecular community,^40^ a second stream of developments emerged, density-based analyses. The interest in these techniques were a result of three main factors: first, density-functional theory originally rests on the density of the system, not its wave function, although—an information often suppressed—the density must be reconstructed using one-electron wave functions, and these usually go under the name Kohn–Sham orbitals. Second, the real-space density is an observable quantity as given by, *e.g.*, electron microscopy and X-ray diffraction, thereby suggesting a more direct footing of any analysis, let alone bonding analysis. Third, the superior efficiency of plane-wave DFT codes for periodic electronic structures did not allow for an atomic decomposition of MO-like wave functions, as all available analyses require LCAO-type molecular orbitals. Hence, traditional bonding analyses from MO theory were inapplicable to the solid state. It is rather unfortunate for any density-based analysis, however, that the density lacks the phase of the wave function which determines bonding or antibonding behavior (the constructive or destructive interference phenomenon recognised by Heitler and London), so the most vital information for any bonding analysis is also lacking. An early example is provided by the so-called Hirshfeld method, dividing the total electron density based on a comparison to the promolecular density, a superposition of atomic densities.^41^ Densities superimpose, only wave functions interfere, as we know since 1927.

The most popular density-based analysis is the so-called quantum theory of atoms in molecules (QTAIM), invented by Bader in 1985.^42^ QTAIM operates *via* the analysis of the topology of the electron density, the latter being chopped into atomic regions by evaluating so-called “zero-flux surfaces” resting on the second derivative of the density. It was soon followed by the so-called electron localisation function (ELF)^43^ defined by an inverse relationship with the like-spins pair probability which decorates (*i.e.*, colours) the density as regards “localised” electron pairs. The course of aesthetically pleasing ELF plots and 3D shapes was quickly shown to be a function of the underlying atomic-orbital topology.^44^ Combinations of ELF and QTAIM have been applied to a wide range of materials to augment chemical bonding analysis^45^ even though ELF, just like the density, does not contain the phases, so it cannot distinguish between bonding, nonbonding and antibonding interactions, easily visible for the 5s4d metal series where neither ELF nor density detect the changing bonding strength upon filling electrons into the bands.^46^ Likewise, increased bonding by *unpairing* spins as in the ^3^O_2_*vs.*^1^O_2_ scenario is hard to imagine using ELF. Eventually, there is the single exponential decay detector (SEDD)^47^ allowing for identification of bonding and non-bonding electrons based on the electron density alone, while antibonding electrons remain elusive to these analyses. These density-based approaches, including ELF, present a topic worth discussion on its own, as other authors already did.^45,48^

On the wave-function front, the ideas of COOP were adjusted to the new Kohn–Sham wave function yielding the Crystal Orbital Hamilton Population (COHP) approach, a COOP successor using DFT.^49^2
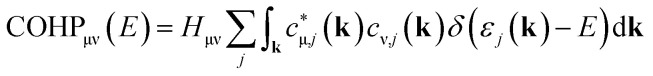


**Fig. 2:**
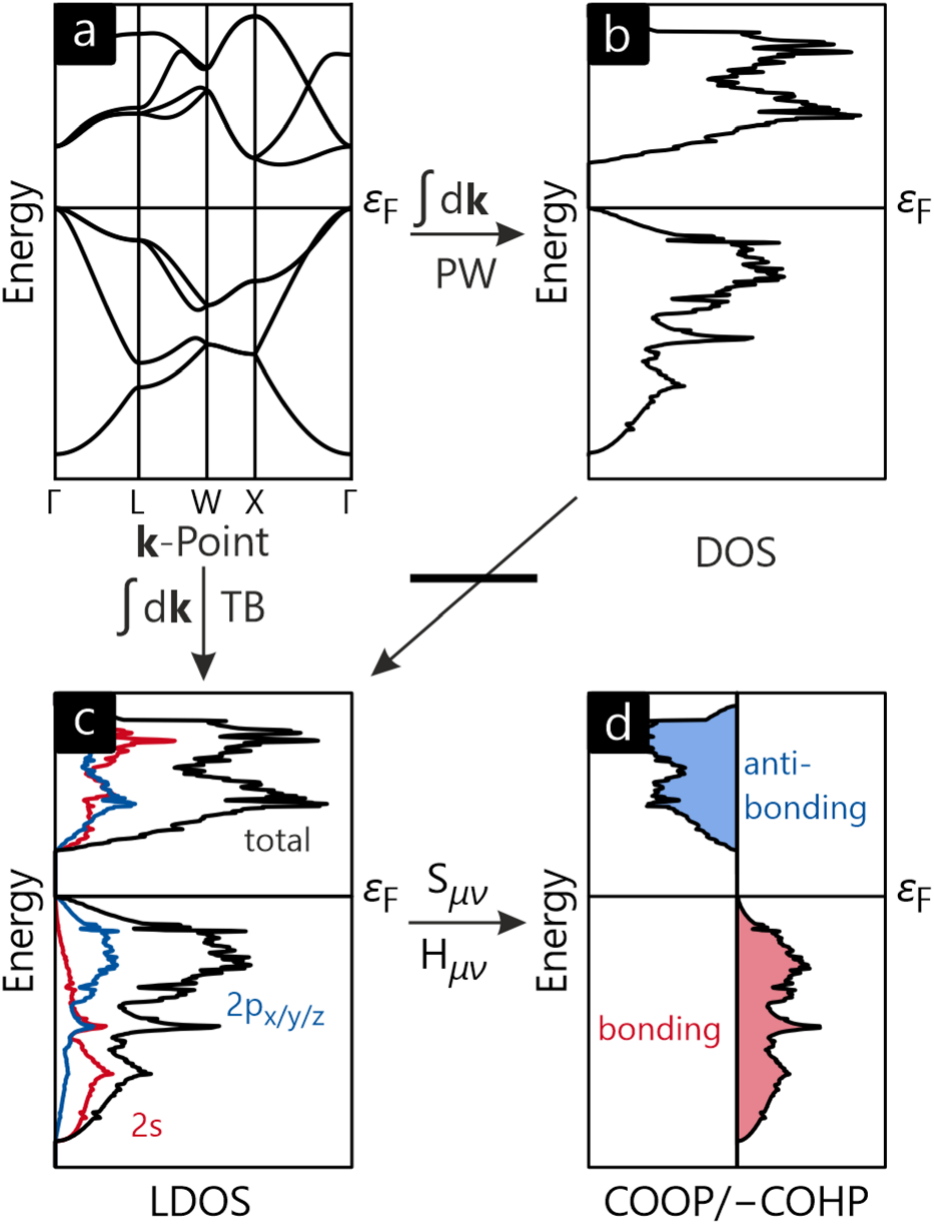
(a) Band structure, (b) density of states, (c) local density of states, and (d) crystal orbital overlap/Hamilton population for diamond. In the context of plane-wave theory, the local DOS, COOP and COHP are not accessible as they require a local-orbital basis, *e.g.*, as provided by a tight-binding model.

The COHP method, state of the art for investigating chemical bonding in periodic electronic structures for at least two decades, was only made possible since an orbital-based theory for band-structure calculations (Linear Muffin-Tin Orbital, LMTO)^50^ was available at that time, also using short-ranged basis sets (TB-LMTO-ASA).^51^ Both COOP and COHP can be pictorially derived as an overlap/Hamilton weighted DOS. Fig. 2 shows the general calculus starting from a band structure (Fig. 2a), diamond in this case. For both plane-wave and tight-binding basis, the band structure can be integrated over the Brillouin zone, resulting in the DOS (Fig. 2b). In case a tight-binding basis is employed, the DOS can be split into individual orbital contributions (see Fig. 2c) and—by multiplication with the overlap integral *S*_μν_ or Hamilton integral *H*_μν_—result in the COOP or COHP, respectively (Fig. 2d). Conventionally, COOP and COHP are plotted such that bonding interactions point to the right-hand side, antibonding points to the left. Hence, we plot positive COOP and negative COHP (a simple graphical convention) because positive overlap and negative energy refer to stabilising (=bonding) interactions.

Quite recently, the Crystal Orbital Bond Index^52^ (COBI) came as a natural extension to COOP and COHP, and COBI is the generalization of the Wiberg and Mayer bond indices for periodic matter and, as such, provides a chemically intuitive quantification of covalent bonds. In addition, COBI allows to look at multi-centre bonds, not only two-centre interactions.

## 2 Lobster

As alluded to before, solid-state chemists need to resort to periodic electronic-structure codes which may come in three different variants for the likewise periodic wave function. To arrive at the latter,^53^ one either starts with numerical partial waves on the individual atoms augmented by plane waves (cellular methods, most accurate), or uses fixed atomic basis functions (tight binding, resembling LCAO), or employs nodeless plane waves (pseudopotential approach).^54,55^ The third approach has turned out so numerically effective that the vast majority of today's DFT simulations is carried out with it. Hence, the plane-wave pseudopotential approach has grown to be a community standard, evident from the large number of citations on codes like VASP^56–59^ or Quantum ESPRESSO.^60–62^ Local chemical-bonding analysis, however, was practically impossible to perform using delocalised plane waves for many years, but this limitation was lifted in 2011 (ref. 9) when the foundations of the LOBSTER code, eventually published in 2013,^10^ were introduced, allowing to seamlessly switch between plane-wave and atomic-orbital bases as well as between reciprocal and real-space representation of electronic structures *via* analytical projection techniques.^63,64^

In LOBSTER, everything is achieved by first reading the plane-wave wave function information from widely used codes such as VASP, Quantum ESPRESSO, or ABINIT (and, since LOBSTER 5.0, also from any electronic-structure code). Afterwards, the overlap integrals between the plane-wave wave functions and predefined contracted all-electron Slater-type orbitals are evaluated in order to generate a transfer matrix, **T**, capable of transferring the information of the periodic electronic wave function, encoded by a plane-wave coefficient matrix **C**^PW^(**k**) to a chemically intuitive atomic-orbital basis without loss of electrons.

3 **C**^AO^(**k**) = **T**(**k**) **C**^PW^(**k**)

The coefficient matrix in the AO basis, **C**^AO^(**k**), can now be used in whatever chemical-bonding analyses discussed in the preceding section. Methods like COHP additionally need the Hamilton matrix in the same basis, which can be reconstructed from the band eigenvalues by a simple unitary transformation:

4 **H**^AO^(**k**) = **C**^AO^(**k**) **E**^PW^(**k**) **C**^AO†^(**k**),

where **E**^PW^(**k**) is a diagonal matrix containing all band eigenvalues at point **k** belonging to reciprocal space. This procedure ensures that the wave function (expressed by the coefficients **C**^AO^(**k**)) and potential (expressed by the Hamilton matrix **H**^AO^(**k**)) are self-consistent, a property of paramount importance in the context of DFT and post-DFT analyses. The quality of the projection is typically evaluated by an overlap criterion, the so-called spilling factor, which gives an overview on how well the exact form of the wave function could be recovered. Typically, spilling factors of single-digit percentages are found, testifying an almost exact recovery of the original wave function, and this also guarantees that all electrons are quantitatively recovered, give or take one thousandth of an electron for a large simulation.

**Fig. 3:**
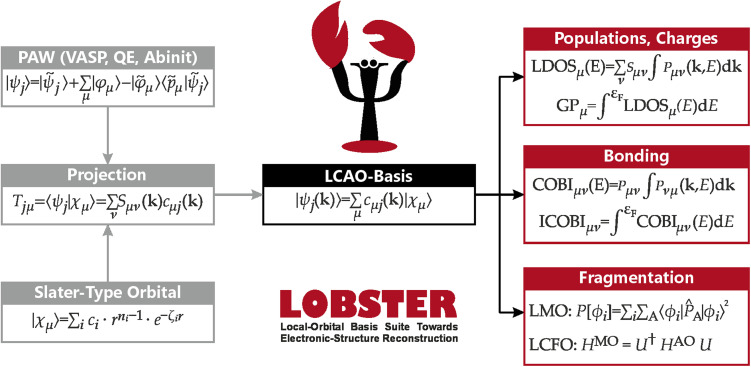
Overview over LOBSTER's workflow. Starting from a PAW plane-wave function and predefined Slater-type orbitals, an LCAO-type wave function is obtained through unitary transformation. This LCAO-type wave function can be used in a large variety of bonding analyses.

The resulting mathematical objects are now suitable for all orbital-based tools mentioned in the preceding section, as schematically shown in Fig. 3. In fact, LOBSTER naturally implements DOS, COOP, COHP, and also COBI, as these solid-state bonding analyses are the bread and butter of its users. Mulliken and Löwdin population analyses have been implemented in 2019,^65^ and Wiberg and Mayer bond indices are available through the COBI implementation.^52^ Localised MOs (LMOs) have been made accessible to the solid-state community for the first time in LOBSTER in 2023,^66^ and fragment molecular orbitals (FMO) have been applied to periodic DFT calculations in 2024 *via* the Linear Combination of Fragment Orbitals (LCFO)^67^ technique, made available to the scientific community with LOBSTER 5.1. Thus, every major development in wave function-based bonding analysis of the past century is now accessible through LOBSTER and can be used by our scientific colleagues around the world, free of charge. In the course of this perspective we will guide the reader through the different orbital-based descriptors currently implemented into LOBSTER.

To do so, it would be natural to showcase the LOBSTER capabilities by a collection of differently bonded solid-state materials, say, metals, semiconductors, oxides, and so forth,^52,68^ or by grouping them into different bonding categories.^46^ On purpose, we here chose a different strategy in which everything will rest on just three compounds, carefully selected. There is the recently identified crystal structure of carbonic acid,^69^ representing a “molecular” crystal containing H bonds. Next, we look at the crystal structure of sodium hydrogen carbonate,^70^ a system comprising both ionic but also H bonding. Finally, sodium carbonate^71^ will serve as another study case, an ionic crystal still containing the complex carbonate anion in which there are covalent bonds. And let us now aim at demonstrating how much can be learned about a compound in just a single LOBSTER calculation.

## 3 Charge analysis

We start the analysis of our study cases with orbital populations, local DOS and charges that do not necessarily allude to (covalent) interatomic bonding but, instead, mirror the consequences of the bonding in terms of “atomic” properties, hence we regard them as “one-centre” bonding indicators. The local density of states (DOS) is calculated using eqn (5) and the total DOS consists of the sum over all local DOS.5
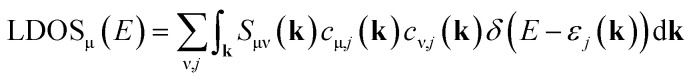
6
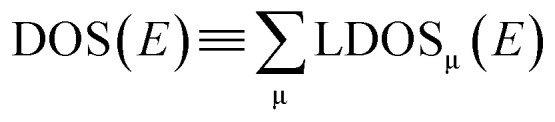


**Fig. 4:**
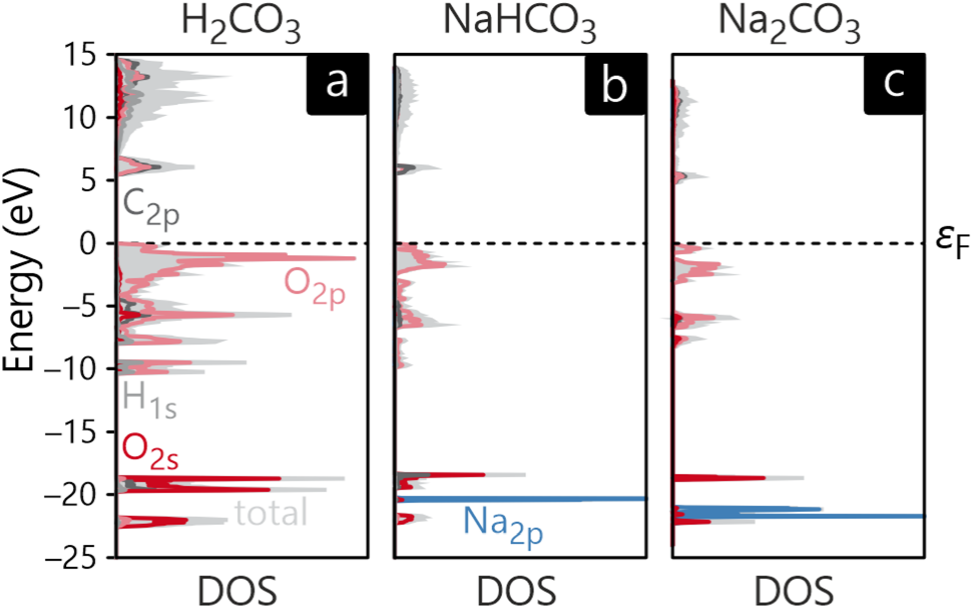
Total density of states as well as the most significant atomic contributions for (a) H_2_CO_3_, (b) NaHCO_3_ and (c) Na_2_CO_3_.

The resulting DOS plots are presented in Fig. 4, showing the total DOS in light grey and the most significant orbital contributions highlighted in other colors.

The occupied valence bands below the Fermi level *ε*_F_ can be described best by viewing them as consisting of two parts in which the lower one approximately ranges from −23 to −18 eV. In the case of H_2_CO_3_ this part is mainly composed of oxygen 2s orbitals shown in red mixing with the C 2p (dark grey) and H 1s (light grey) orbitals, contributing to the covalent (σ) backbone of the molecule. The contributions from the O atom, however, clearly predominate here. In the cases of NaHCO_3_ and Na_2_CO_3_ the Na 2p contributions shown in blue are the largest in this energy region, reflecting filled and sharp semi-core levels, not engaged in chemical bonding. The virtual, unoccupied Na 3s levels, on the other hand, are way above the Fermi level, not given in the figure. Right below the Fermi level, the O 2p orbitals form the valence band maximum in all three systems by mixing with the C 2p (also generating π bonding) and H 1s orbitals. Within the unoccupied conduction band, the 2p orbitals of C predominate.

The analysis of the density of states can be further complemented by the orbital gross populations which are calculated using the approaches introduced by Mulliken^21–24^ and Löwdin.^25^ Following Mulliken's technique, the gross population (GP_μ_) is determined *via* the density-matrix formalism given in eqn (7). Alternatively, one may formulate the gross population as an integral of the corresponding local DOS to the Fermi level.7
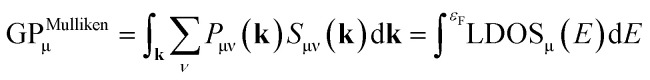


*P*_μν_(**k**) and *S*_μν_(**k**) are the **k**-dependent density and overlap matrix elements for the orbitals μ and ν. The density matrix element is computed from the LCAO coefficients *c*_μ,*j*_(**k**) in reciprocal space as demonstrated in eqn (8) whereas *S*_μν_(**k**) depends on the basis functions *χ*_μ_(**k**).8
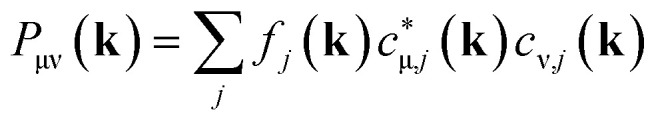

9 *S*_μν_(**k**) = 〈*χ*_μ_(**k**)|*χ*_ν_(**k**)〉

Upon applying Löwdin's symmetrical orthonormalization (LSO), a new density matrix *P*′ = *S*^−½^*PS*^−½^ is obtained, and this then leads to the Löwdin gross population having the following form10
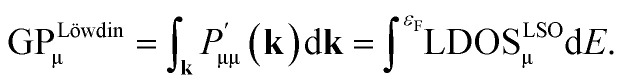


The numerical results of both Mulliken and Löwdin population analyses for H_2_CO_3_, NaHCO_3_ and Na_2_CO_3_ are presented in Tables 1 and 2.

**Table 1:** Mulliken gross orbital populations for H_2_CO_3_, NaHCO_3_, and Na_2_CO_3_

Atom	Orbital	H_2_CO_3_	NaHCO_3_	Na_2_CO_3_
C	2s	0.71	0.74	0.79
	2p	0.80	0.81	0.83
O	2 s	1.72	1.73	1.74
	2p	1.61	1.65	1.69
H	1s	0.55	0.59	—
Na	2p	—	2.00	2.00
	3s	—	0.12	0.16

**Table 2:** Löwdin gross orbital populations for H_2_CO_3_, NaHCO_3_, and Na_2_CO_3_

Atom	Orbital	H_2_CO_3_	NaHCO_3_	Na_2_CO_3_
C	2s	0.76	0.77	0.79
	2p	0.76	0.86	0.87
O	2s	1.56	1.60	1.62
	2p	1.62	1.65	170
H	1s	0.65	0.67	—
Na	2p	—	2.00	2.00
	3s	—	0.27	0.26

A short analysis of these population data yields that similar trends can be deduced from both, not too surprisingly. On carbon and oxygen, respectively, the 2s and 2p orbitals are almost equally populated which, in an organic chemist's terminology, can easily be explained by significant orbital mixing (or hybridization), mirroring the special role of the first long period^46^ (also going under the term primogenic repulsion).^72^ It is also worth mentioning that upon increasing Na content the population of all orbitals (except for Na 3s in the Löwdin approach) is increased since more electron density is dumped on the complex anion by the least electronegative atom, Na.

From the gross orbital populations it is only a short path to the calculation of wave function-charges. The Mulliken or Löwdin charges *q*_A_ for an atom A are formulated as the difference of the valence electrons *N* and the sum of the gross orbital populations of all orbitals μ on atom A.11
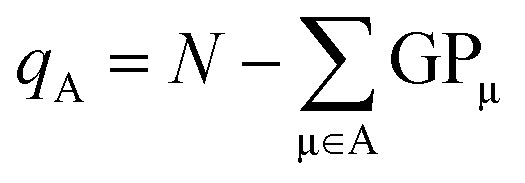


**Fig. 5:**
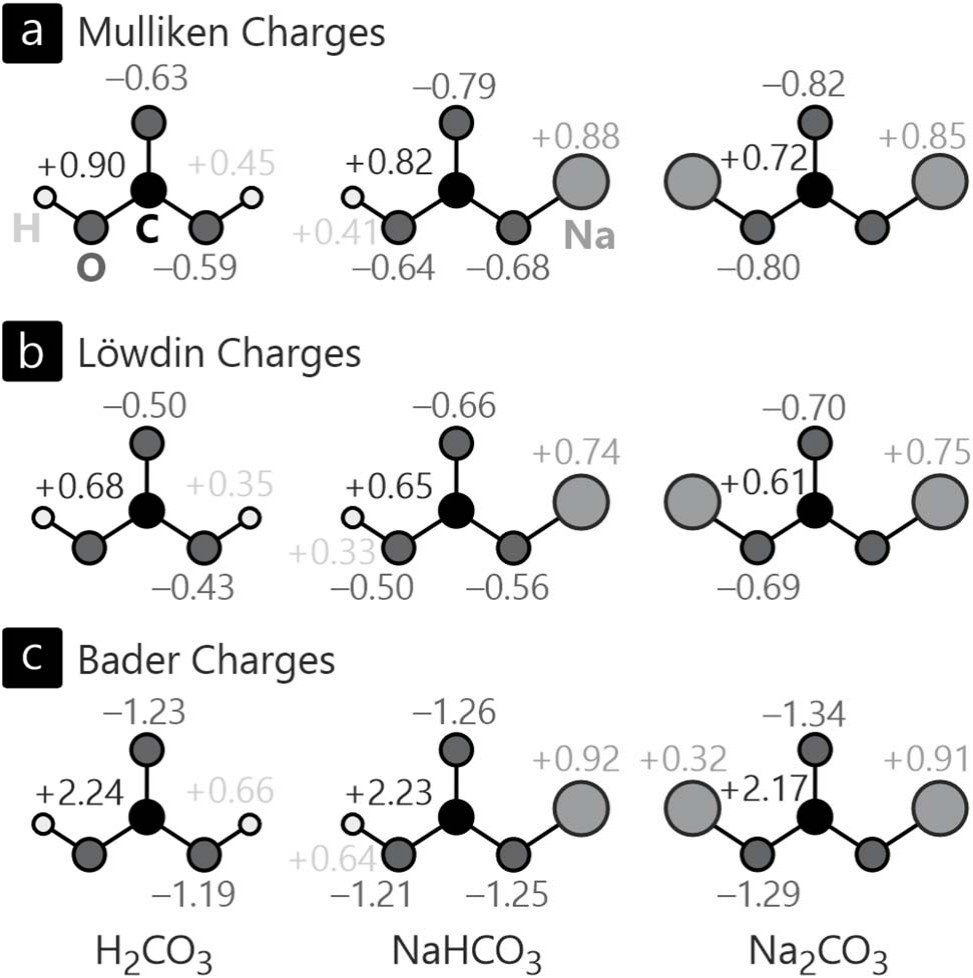
Simplified depictions of solid-state carbonic acid, sodium hydrogen carbonate and sodium carbonate with the corresponding (a) Mulliken and (b) Löwdin charges as calculated using LOBSTER. Bader charges are provided in (c) for comparison.

The charges computed according to eqn (11) for H_2_CO_3_, NaHCO_3_, and Na_2_CO_3_ are depicted in Fig. 5a and b. In addition, so-called Bader charges are included in (c) for comparison with a density-based approach. As alluded to already, Bader's calculus^42,73–75^ partitions the electron density into atomic regions using a topology criterion. These so-called basins are determined *via* the zero-flux condition given in eqn (12) where *ρ*(**r**) is the electron density at any point **r** on the surface with the normal vector **n**(**r**). Once the atomic basins have been determined, Bader charges can be calculated analogous to Mulliken and Löwdin populations, not from the (unavailable) wave function but from the density.

12 ∇*ρ*(**r**)·**n**(**r**) = **0**

One glance at Fig. 5 immediately reveals that the charges obtained using orbital-based approaches (a and b) differ rather significantly from those obtained using Bader's density-based technique (c). Nonetheless, with increasing Na content the charge transfer is increased, especially between O and Na, a consequence of their very different electronegativities.

This increasing ionicity is not only reflected from the charges but also from the Madelung energies since, in a crystalline solid, ionicity straightforwardly leads to the so-called Madelung field and an (electrostatically defined) lattice energy.^76^ The purely electrostatic part of the lattice energy, *i.e.*, the Madelung energy, is automatically calculated in LOBSTER based on Mulliken or Löwdin charges. The results for all three study cases are given in Table 3. All of them arrive at large values, even for a molecular crystal like carbonic acid, on the order of rock salt. Nonetheless, these—rather fictitious—Madelung energies become more negative with increasing sodium content which clearly indicates the increasing ionicity of those systems.

**Table 3:** Madelung energies *ε*_M_ based on Mulliken and Löwdin charges for H_2_CO_3_, NaHCO_3_, and Na_2_CO_3_

System	*ε*_M_^Mulliken^ (kJ·mol^−1^ )	*ε*_M_^Löwdin^ (kJ·mol^−1^ )
H_2_CO_3_	−1807	−1042
NaHCO_3_	−2108	−1401
Na_2_CO_3_	−2356	−1763

Coming back to the comparison between orbital- and density-based charges, the trend seen for the three compounds is mirrored by the atomic charges obtained with all three approaches, although the Bader charges arrive at much larger values (more than twice) compared to Mulliken and Löwdin charges. Such stronger emphasis on electron transfer is rather typical for the Bader approach, a consequence of chopping the density.

Bader's larger absolute values of the net charges^77^ therefore paint a more ionic picture of the structures under investigation which is closer to what is expected from the classical oxidation state, mirroring the semi-classical methodology lacking the wave function. As mentioned earlier, Bader cuts the electron density based on topology into non-overlapping domains, as if there were no overlap (as in ionic compounds). In compounds like those treated in this perspective, however, it is obvious that covalency is at play and raises concerns whether the treatment as non-overlapping domains is at all suitable. We recall that the covalent bond is an interference phenomenon of overlapping wave functions, exactly that. For carbonic acid, the covalent character of the bonds has already been identified in an earlier work by some of the authors.^69^ In the following analyses, we will further discuss and evaluate the ionic picture painted by the Bader charges for the strongly covalent systems under investigation.

## 4 Bond orders

While the “ionicity notion” has prevailed in solid-state chemistry for many decades (even for GaP, see above), the reason being that quantum-chemical approaches were simply unavailable, hence Madelung arguments are found in virtually every solid-state chemistry textbook, and we did the same in the preceding section on purpose. The situation in molecular chemistry, in particular organic chemistry is very different. Here, covalent bonding is the norm almost playfully. Formation and cleavage of, *e.g.*, C–C bonds is regularly used to understand and predict experimental phenomena such as reactivity and stability. These relations of chemical bonding are backed even more so by quantum mechanics and orbital theory, and the Woodward–Hoffmann rules serve as a fitting example,^19^ mirroring the fact that the covalent bond originates from interfering wave functions. Even though an ionic notion then seems totally counterintuitive for a molecule, the strength of a covalent bond may be quantified by considering the atomic valence, borrowing structural ideas from Pauling.^17^ The method going under the name bond length-bond strength is fairly mature, and in this context let us highlight a modern descendent, the empirical bond valence sum^78^ (BVS) recipe which correlates the bond order with the interatomic distance, given that a plethora of experimental data is available. While this kind of analysis serves well as semi-quantitative measure, it is essentially non-quantum-chemical and does not reflect the underlying orbital overlap or symmetry. The latter consideration requires a wave function based on, say, atomic orbitals,^24,79^ plane Bloch-waves,^80,81^ or maximally localised Wannier functions.^29^

One method to calculate orbital-based bond orders was published 2021 by the authors in the framework of the LOBSTER package. In the spirit of Hughbanks' and Hoffmann's COOP approach involving the overlap and density matrices, the original molecular bond index by Wiberg^30^ and Mayer^31,32^ was generalised to the solid state and dubbed crystal orbital bond index (COBI) as shown in eqn (13).^52^13
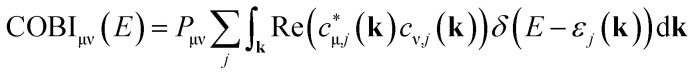
Here, elements of the density matrix show up twice. In contrast to COOP (an overlap measure) and COHP (an energy measure), the intuitive analysis of COBI provides an even lower activation barrier as its energy integral, dubbed ICOBI, equals the covalent bond order that is covered in any basic chemical lecture and relates to Lewis-style molecular sketches involving one, two, three…dashes between various atoms, *e.g.*, C–C, O=O, N≡N, *etc.* Coming from a density-centred analysis, such as QTAIM, a covalent bonding analysis is not directly possible but requires an auxiliary wave function (which then delivers phases). Based on the latter, so-called delocalisation indices (DI) are calculated from the overlap *S*_*i*,*j*_ between the auxiliary wave functions *ϕ*_*i*_ and *ϕ*_*j*_ integrated over the QTAIM basins Ω_A_,^82^ so the density-based approach is no longer exclusively focusing on the density.14
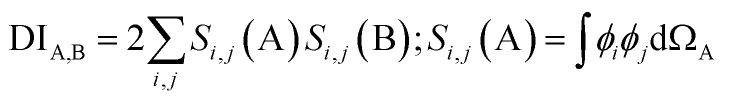


**Fig. 6:**
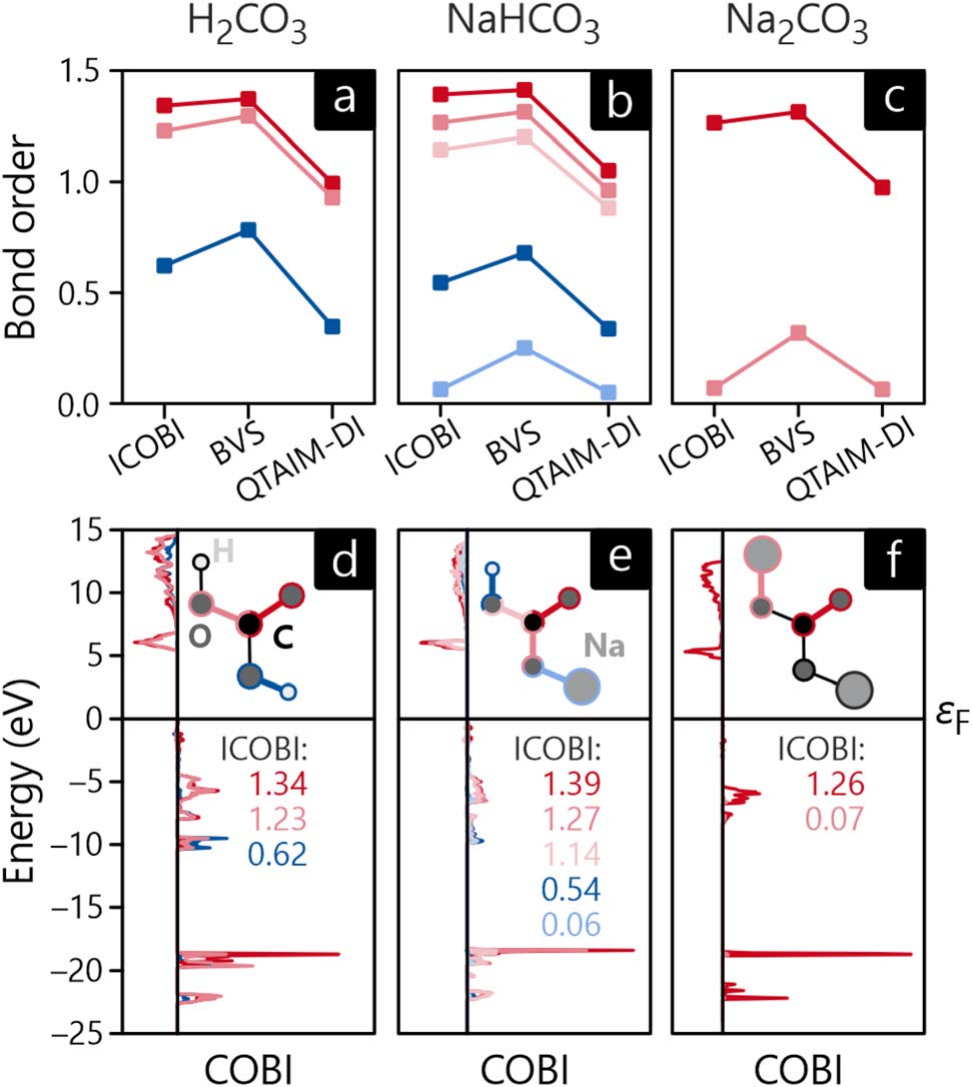
Bond orders of (a) H_2_CO_3_, (b) NaHCO_3_ and (c) Na_2_CO_3_ as well as energy-resolved crystal orbital bond indices of (d) H_2_CO_3_, (e) NaHCO_3_ and (f) Na_2_CO_3_. The color codes of the bond orders in (a)–(c) correspond to the color-coded bonds in (d)–(f).

Fig. 6a–c shows the bond orders of various bonds in H_2_CO_3_, NaHCO_3_, and Na_2_CO_3_, either calculated quantum-chemically with ICOBI, empirically (or classically) by the BVS approach, and also density-based including auxiliary orbitals using QTAIM-DI. A quick inspection of the values for the C–O (red curves in Fig. 6) as well as the O–H bonds (dark blue curves) reveals a striking similarity between quantum-chemical ICOBI and empirical BVS in that all C–O bond orders lie between 1.23 and 1.42 with only minor differences between both fundamentally different methods. This correlation comes to no surprise as C–O bonds have been widely studied in organic chemistry, hence forming an extraordinarily large and also reliable reference database for the empirical BVS parameters. The QTAIM-DI values, on the other hand, are lower than the single bond order in all cases which requires explanation. While both ICOBI and QTAIM-DI rely on a wave function, the latter only indirectly, the partitioning is quite different in both methods. QTAIM cuts the electron density based on topology, resulting in non-overlapping domains by definition. Such a treatment may raise concern in strongly covalent materials because—again—the covalent bond is an interference phenomenon of overlapping wave functions. In contrast, a likewise simplifying approximation does not apply when atomic orbitals are employed for the calculation of ICOBI, so the resulting bond orders are more in line with the empirical expectations. The Na–O bonds show a different trend, however: in this case, the bond orders from ICOBI and QTAIM-DI are extremely small for both NaHCO_3_ and Na_2_CO_3_, and the empirical BVS arrives at about 0.3 in both compounds, a number that must be considered even qualitatively incorrect. The underlying reason is easily found in the bonding mechanism of these contacts, namely ionicity, mirroring the large EN difference between oxygen and sodium. Both compounds feature Na^+^ cations that do not form any covalent bonds due to their closed-shell nature, so the amount of covalency can only be very minor, in harmony with both QTAIM-decomposition as well as ICOBI (projection to atomic orbitals) which arrive at similar results. The empirical BVS overestimates the covalent strength since a change in bonding mechanism (covalency *vs.* ionicity) is not built in, the simple parametrization does not reflect it, and even grossly different oxidation states, *i.e.*, Na^+^, Na^0^, and Na^−^, would be treated equally despite different valence-electron numbers, different atomic sizes, *etc.*

One key difference between (I)COBI and BVS or QTAIM-DI discussed before is its energy-as well as orbital-resolved analysis, similar to preceding methods such as COOP and COHP because they rely on an energy-resolved density-of-states matrix including entries from all atomic orbitals. Hence, Fig. 6d–f shows the energy-resolved COBI plots for all previously discussed bonds in H_2_CO_3_, NaHCO_3_, and Na_2_CO_3_. A glance at these evidences that the bonding in all three compounds occurs mainly within energy levels around −5, −10, and −20 eV. Second, the levels close to the valence-band maximum, directly below the Fermi level, appear as essentially nonbonding. Not too surprisingly, the levels generating the unoccupied conduction bands reflect antibonding interactions throughout, as expected from basic MO-theory.^79^

**Fig. 7:**
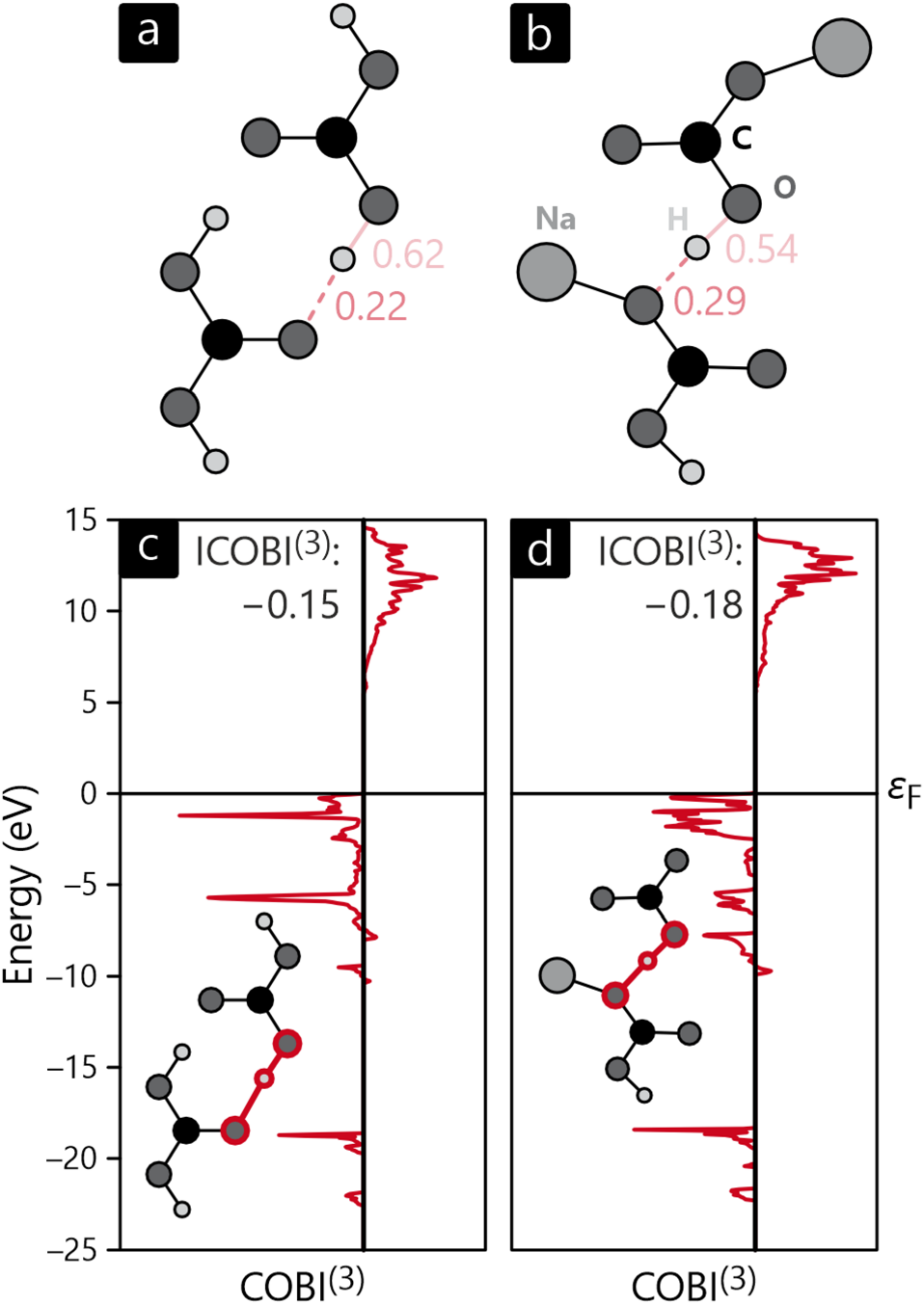
Sketches of intermolecular interactions (and corresponding bond orders) in the crystal structures of (a) H_2_CO_3_ and (b) NaHCO_3_ as well as the energy-resolved three-centre bond index, COBI^(3)^, for (c) H_2_CO_3_ and (d) NaHCO_3_.

The bonding analysis thus far focused on intra-molecular bonds, the by far strongest interactions, but the cohesion of a molecular crystal, however, arises from interactions *between* molecules, in inter-molecular bonds. In the present examples, hydrogen-bonding can clearly be identified as the driving force for the condensation of H_2_CO_3_ and NaHCO_3_, the latter compound also including ionic interactions stemming from the Na^+^ cations, see above. Fig. 7a and b visualise the intra- and inter-molecular O–H-bonds for both compounds and their bond orders as determined with COBI. The shorter (intra-molecular) O–H bonds possess larger bond orders of 0.62 and 0.54, indicating significant covalency in these contacts. The longer bonds would typically be described as H-bonds or H-bridges but clearly mirror a certain covalency with ICOBI of 0.22 and even 0.29 for NaHCO_3_, in harmony with hydrogen-bond covalency quantified before.^83^ Interestingly, the sum of both bonds, inter- and intra-molecular, is about 0.83 for both cases, meaning that the overall bonding capacity is comparable and only shifted differently to the individual bonds, suggesting some form of cooperativity, that is, questioning whether this sort of bonding can be described as two distinctive bonds or if a collective picture is more appropriate. An answer can easily be found using a multi-centre bonding analysis^84–86^ that is accessible with COBI^(3)^—the superscript symbol indicating three-centre interactions—as given in eqn (15).15
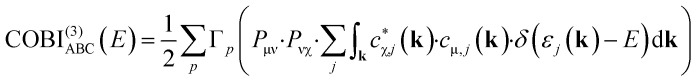


Multi-centre interactions are well known from metals, of course, defining how covalency turns into metallicity if too few electrons must be distributed over a plethora of atoms, namely by occupying a wave function that encompasses more than two atoms, possibly even an infinite numbers atoms. In the present molecular case, the situation is not that extreme, as exemplified by the COBI^(3)^ plots of the O–H–O three-centre bonds shown in Fig. 7c and d. Clearly, three-centre interactions are found in both compounds, and they span the entire occupied region, as seen from the red curves. Another important detail relates to the negative sign of the energy-resolved COBI^(3)^ as well as the energy-integrated ICOBI^(3)^. Negative numbers typically occur for electron-rich three-centre bonding and have been interpreted as such^46^ but we note that similarly negative values have also been found for H-bonding.^87^ Interestingly, the effect of multi-centre bonding is stronger in the more symmetric bond of NaHCO_3_ by about 20%.

On a more technical level, every multi-centre bond index can—in principle—be generalised to any number of centres involved, and the case of metallicity proves the point. In practice, however, one will reach real-world limits due to finite computational power and also error propagation. Additionally, the numerical analysis of higher-order bonding is far from being trivial (except the metallicity extreme) so we will restrict ourselves to two- and three-centre bonding here. Let us recap, however, that such multi-centre-bonding analysis is a consequence of collective orbital interactions beyond classical pairwise bonding, thereby mirroring the quantum character of covalency as a wave function is not bound to two atoms. If such a multi-centre bond index adopts non-zero values, a truly delocalised bond (at least to some degree) is indicated, for example in the electron-rich XeF_2_ or the electron-deficient B_2_H_6_ molecules.^46,52^

## 5 Fragmentation

The previous sections have focused on interactions based on charges and covalent pairwise as well as multi-centre bonding between individual atoms. Yet, chemistry is more than that since molecules or molecular (complex) ions can also be understood as being composed of groups of atoms. Hence, molecular chemists very often divide a large molecule into multiple functional groups that help categorise and generalise the reactivity of the entire molecule. Similarly, crystal chemistry features molecular ions and complex polyhedra that can be described, at least approximately, as almost isolated entities. For example, one may of course understand crystalline Na_2_CO_3_ as being composed of two Na, one C, and three O atoms but a more chemical notion of two Na^+^ cations and one CO^2−^_3_ carbonate anion is far more fitting, alluding to the fragments showing up in aqueous solution chemistry.

Such a fragmentation analysis is also part of the quantum-mechanical toolkit provided by the LOBSTER package. In order to extract a localised fragment from a delocalised reciprocal-space Bloch wave function, the wave function needs to be transformed into real space. Using a so-called embedding approach,^88,89^ the total density *ρ*^tot^ is split into an active part *ρ*^act^ and an environmental part *ρ*^env^. Therein, the active density is calculated explicitly whereas the environmental density acts as an external potential onto the active one. For the exact details we refer the interested reader to the literature.^88,89^

16 *ρ*^tot^(**r**) = *ρ*^act^(**r**) + *ρ*^env^(**r**)

**Fig. 8:**
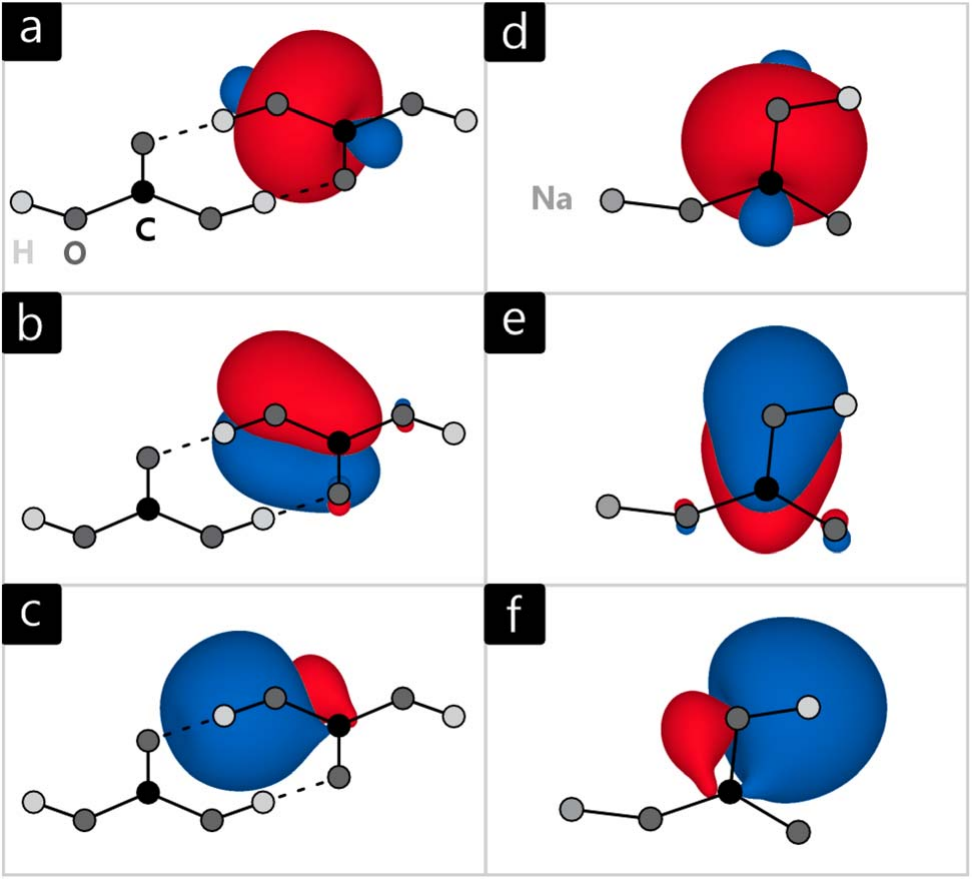
Localised molecular orbitals of (a–c) H_2_CO_3_, and (d–f) NaHCO_3_, indicating σ bonds (top), π bonds (middle), and H bonds (bottom).

Once the environment density has been generated, it is then used to calculate the wave function of the active system in real space which finally can be analysed in an analogous manner to isolated molecules. For this, we implemented a Pipek–Mezey^28^ algorithm into LOBSTER that generates Localised Molecular Orbitals^66^ (LMO) by maximising the Mulliken gross population *P* of the molecular orbitals *ϕ*_*i*_:17
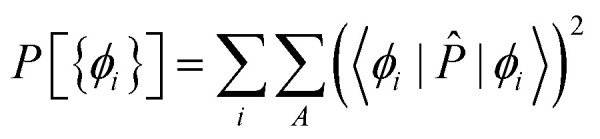
with18
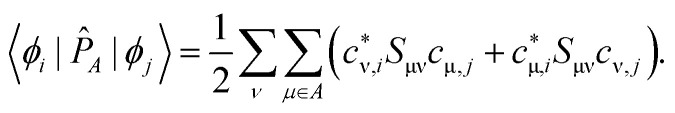
The orbitals generated by this procedure are written out for further analysis. Fig. 8 exemplifies a selection of LMO for H_2_CO_3_ and NaHCO_3_, to be separated into three categories: Fig. 8a and d depict σ-bonding orbitals of C–O bonds in both molecules while Fig. 8b and e includes π-bonds, energetically on top of the σ-orbitals. Fig. 8c and f visualises bonds involving H-atoms. As these orbitals extend beyond hydrogen, they can be attributed to the hydrogen bonds discussed in the previous section, perfectly in line with their multi-centre character.

An alternative theory was introduced in the same LOBSTER framework. Based on the fragment molecular orbital method^34,90^ as developed with extended Hückel theory decades ago, the Linear Combination of Fragment Orbitals (LCFO)^67^ follows essentially the same idea, this time based on a first-principles approach and involving periodic boundary conditions. Similar to the LMO technique discussed before, LCFO uses a Fourier transform of the delocalised wave function to real space according to eqn (19).19
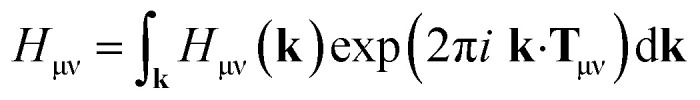


This real-space Hamiltonian is then diagonalised following eqn (20), yielding the unitary transformation matrix **U** including the LCAO coefficients of the local atomic orbitals forming the local molecular or fragment orbitals.

20 **H**^AO^ = **UH**^MO^**U**^†^

The definition of LCFO is flexible enough to involve multiple fragments of any composition. Eqn (21) shows the construction of an overall transformation matrix **U**_total_ from two distinct fragments 
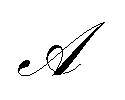
 and 
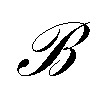
. This total matrix is a block diagonal matrix containing the individual matrices 
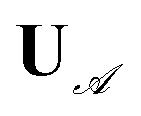
 and 
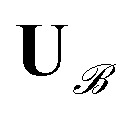
 on its diagonal whereas all other elements are zero.21
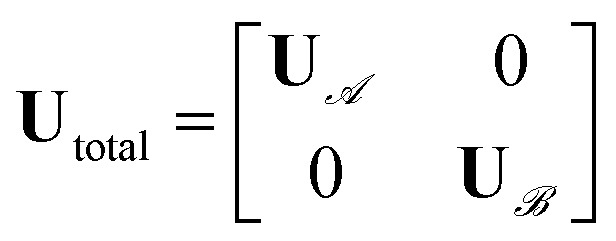


This total transformation matrix is then used to transform the atomic-orbital basis in reciprocal space into a fragment-orbital basis. Eqn (22)–(24) cover the transformation of coefficient, Hamilton and density matrices. For technical reasons, the overlap matrix is not available for analysis, on purpose, as the LOBSTER fragmentation is performed for an orthonormal basis.

22 **c**^MO^_total_(**k**) = **U**^†^_total_**c**^AO^_total_(**k**)

23 **H**^MO^_total_(**k**) = **U**^†^_total_**H**^AO^_total_(**k**) **U**_total_

24 **P**^MO^_total_(**k**) = **U**^†^_total_**P**^AO^_total_(**k**) **U**_total_

With this new fragment-orbital basis, we can now use LOBSTER's complete toolkit that is available for the atomic-orbital basis, *i.e.*, DOS, COHP, and COBI.25
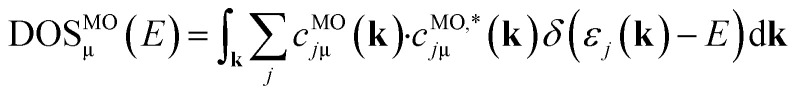
26
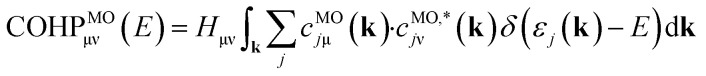
27
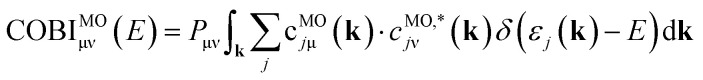


**Fig. 9:**
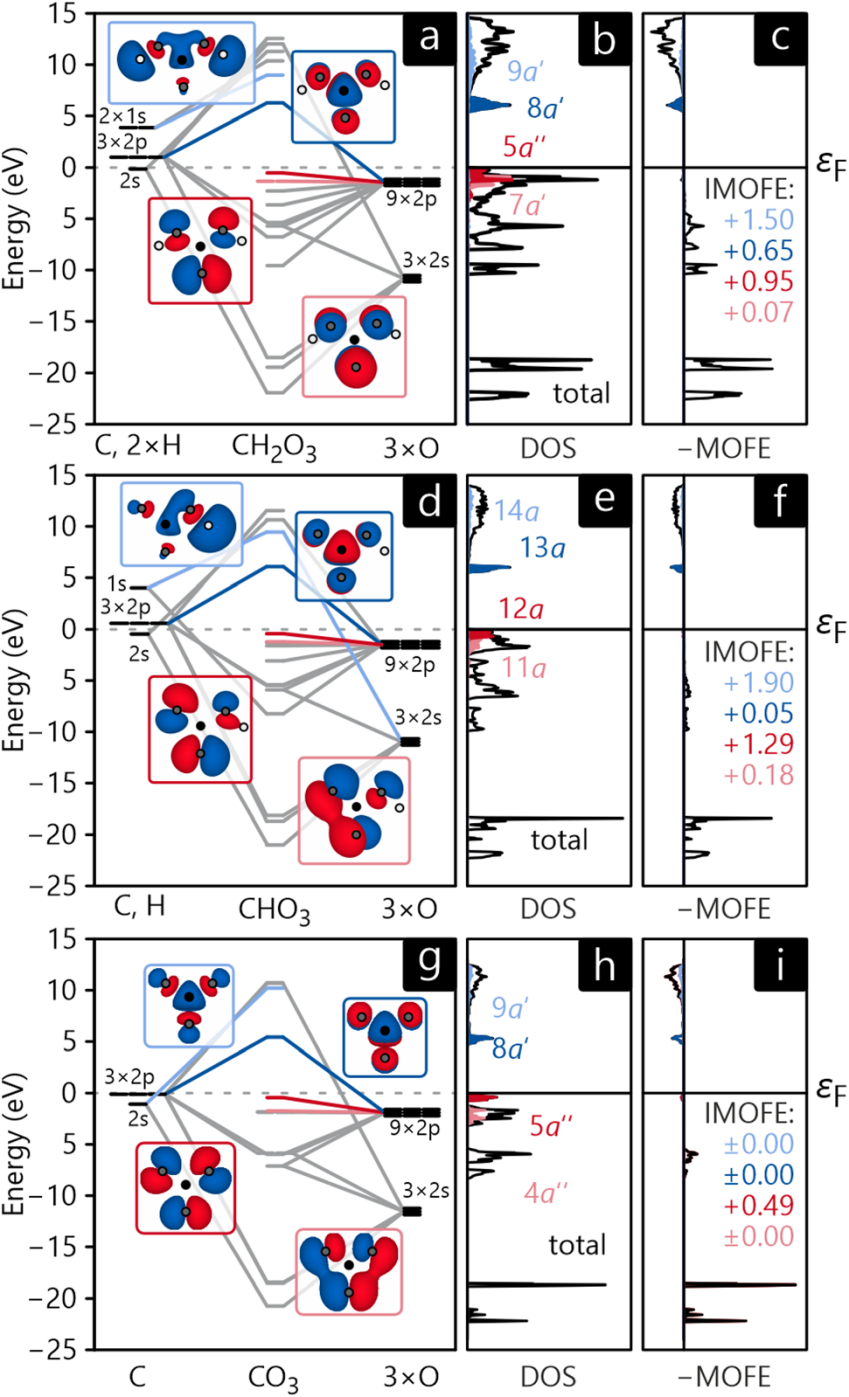
Molecular orbital diagram (a, d and g), MO-projected density of states (b, e and h) and molecular orbital formation energy MOFE (c, f and i) for H_2_CO_3_ (a–c), HCO^−^_3_ (d–f), and CO^2−^_3_ (g–i). IMOFE is given in eV.

The MO diagrams and DOS in the MO basis of crystalline H_2_CO_3_, NaHCO_3_, and Na_2_CO_3_ are shown in Fig. 9a, b, d, e, g and h. Unsurprisingly, the molecular orbitals of all three crystals are very similar in shape and have the same energetic trends, corroborating the chemical concept of viewing all three compounds as H/Na-bonded variants of an carbonate anionic core. The HOMO of all compounds (shown in dark red in Fig. 9) has the shape of the carbonate anion and essentially consists of p-orbitals of oxygen while a quick visual inspection identifies them as non-bonding. The LUMO (marked in dark blue in Fig. 9) is formed by an antibonding combination of p-orbitals, however, residing on carbon and oxygen perpendicular to the anion's plane. The DOS curves reveal that HOMO and LUMO form valence and conduction bands, respectively, for all three compounds. These findings are in perfect agreement with the atomic orbital-based DOS shown in section 3, not too surprising as both are calculated from exactly the same electronic structure.

As an additional tool, let us introduce the molecular orbital formation energy (MOFE) which quantifies the band-energy contribution of an atomic orbital μ towards the formation of a molecular orbital α. Depending on the exact question to be answered, MOFE either measures the total contribution of an atomic or molecular orbital to the overall molecule or—if a more detailed analysis is required—it can be broken down into individual μ–α contributions.28
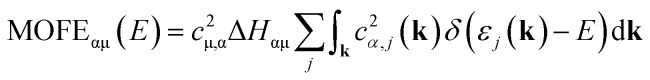


For illustration, the MOFE plots in Fig. 9c, f, and i allow a very detailed analysis of the molecular orbitals. Interestingly, the IMOFE of all orbitals explicitly shown in this analysis has a positive sign, meaning that these orbitals destabilise the molecule to a certain extent as they increase the total energy. Naturally, this destabilisation is more than counteracted by bonding interactions at lower energies not shown explicitly. As the absolute values of all IMOFE are rather small, however, it is safe to designate these molecular orbitals as essentially nonbonding (as said before), so phrasing them as “lone pairs” looks reasonable. For reference, a C–C single bond in diamond has a stabilising effect of about −9.66 eV to the one-particle band-structure (Kohn–Sham) energy, not to be confused with an experimental C–C single-bond dissociation enthalpy of about 348 kJ mol^−1^.^91^

**Fig. 10:**
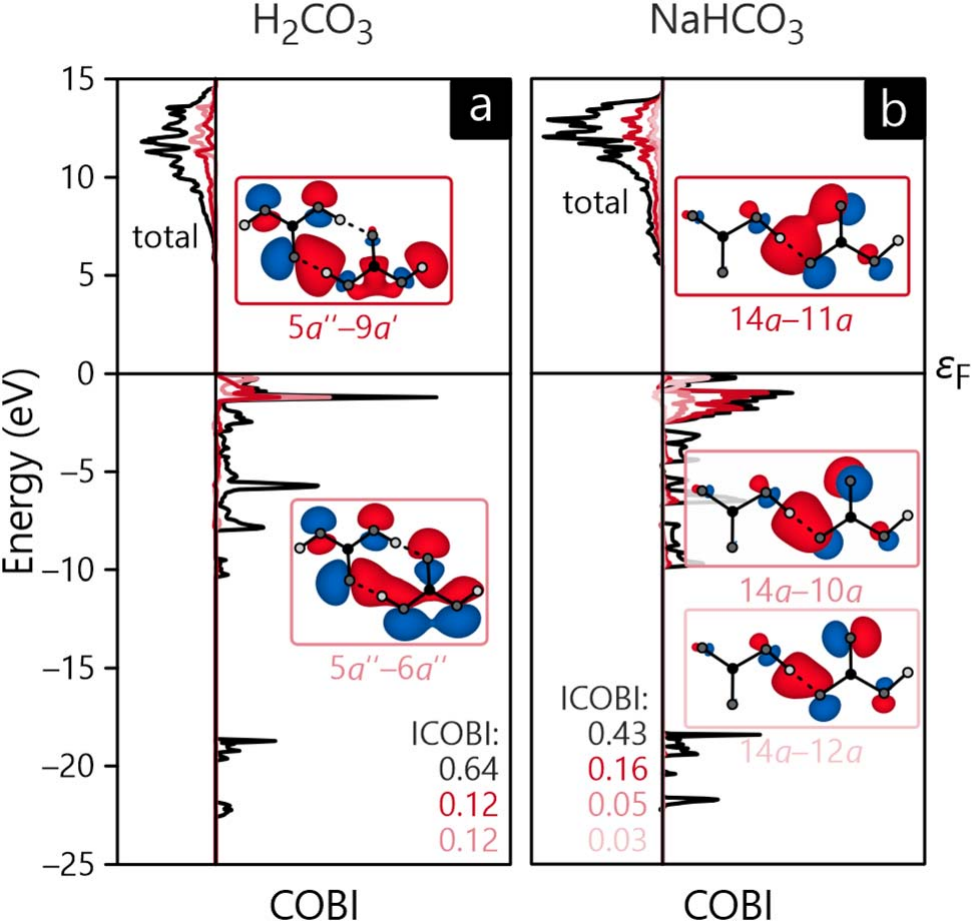
Crystal orbital bond index diagram of (a) a H_2_CO_3_–H_2_CO_3_ bond in carbonic acid and (b) a HCO^−^_3_–HCO^−^_3_ bond in NaHCO_3_. The color-coded orbital–orbital interactions with the highest contribution are shown explicitly.

As already alluded to before, the LCFO method can also be used to calculate interactions between molecules. For this consideration, we chose to exclude Na_2_CO_3_ as inter-molecular bonding is primarily ionic in this compound. Hence, Fig. 10 only depicts the COBI diagrams for a H_2_CO_3_–H_2_CO_3_ bond as well as a HCO^−^_3_–HCO^−^_3_ bond. From an atomic-orbital perspective, these bonds have already been identified as hydrogen-bonds, now confirmed by the molecular-orbital-based COBI. Interestingly, the total bond order is larger for H_2_CO_3_ (0.64) than for NaHCO_3_ (0.43), even though the ICOBI of the individual H-bonds indicate the opposite trend but this tentative disagreement is easily explainable by the multiplicity of these bonds in the respective compounds: H_2_CO_3_ adopts a dimeric structure and is involved in two H-bonds per molecule pair whereas NaHCO_3_ only has one such bond, resulting in a lower total bond order. When comparing the ICOBI of the MO basis and the AO basis, we find that the H-bonding accounts for about 68% of the total bond strength, and other more long-ranged effects such as ionicity contribute to the rest.

## 6 Conclusion and outlook

In the course of this perspective we have summarised major developments in chemical bonding analysis ranging from the beginnings in 1916 until the present day, with a special focus on wave function-based approaches. To correctly analyse solid-state (periodic) materials in terms of chemical bonding, the LOBSTER suite makes orbital-based descriptors, known from molecular chemistry, easily available for crystalline matter, too, namely through an accurate analytical (unitary) transformation from the plane-wave into an atomic-orbital picture. Three carbonate study cases known to all readers with a chemistry background have been presented, including currently implemented bonding descriptors which relate to wave function-based atomic charges and pairwise covalent as well as multi-centre bonding. Complementing the atomic-orbital basis, further analyses have been carried out, highlighting local molecular as well as fragment orbitals that allow for a deeper understanding of solids based on concepts from molecular chemistry.

As density-based analytic techniques alluding to chemical bonding have been popular (thanks to their simplicity of calculation) in the solid-state context, the LOBSTER orbital-based results have been compared with descriptors stemming from Bader's QTAIM method. It is clear from the outset that the quasi-molecular solid-state systems investigated here are driven by covalency but charges calculated from Bader's QTAIM approach paint a far more ionic picture than Mulliken or Löwdin charges obtained *via* wave functions (LOBSTER). Likewise, while wave function-based ICOBI values corroborate the expected covalency in the investigated systems, density-based QTAIM-DI yields much lower bond orders. This is unsurprising since QTAIM cuts the electron density into non-overlapping domains, a somewhat unfitting description for covalently bonded systems. Despite such problem, we do not intend to categorically withhold the value of density-based approaches. Alternative partitioning schemes such as Hirshfeld's method^41^ and derived approaches^92–97^ allow the atomic basins to overlap, drawing a picture closer related to wave-function theory. Such an implementation may benefit from the local-orbital framework as provided by LOBSTER's tool set, so a modified Hirshfeld-based population and charge analysis seems promising in this context.

Additionally, one may already now envision other wave function-based extensions to the orbital toolkit, for the betterment of solid-state chemical-bonding analysis. For example, orbital-based descriptors may also include atom/element-specific information, unavailable for any density-based approach, as orbital extent by contraction parameters as a function of effective nuclear charge, for example, are only available in the former. In contrast, orbital-based techniques, typically depicting bonding criteria in an energy-resolved way, may adopt the style known from density-based approaches which drop the (unavailable) energy dependency in favor of a real-space representation, that is, a density map known from, say, X-ray crystallography. In principle, this can also be accomplished using orbital-based methods, so it is under development right now. Taken together, we trust that all those techniques will create further bridges between theoretical and synthetic chemists.

## 7 Computational details

Electronic-structure calculations involving structural optimizations were carried out using the Vienna *ab initio* simulation package (VASP) 5.4 (ref. 56–59) with PAW-based^98^ pseudopotential-like electronic wave functions. As H_2_CO_3_ is a high-pressure phase and comparison with the two other compounds had to be performed, an external pressure of 2 GPa was applied to all three structures during optimization. The simulations were considered converged for energetic differences of 10^−8^ eV for electronic and 10^−6^ eV for ionic optimizations. The kinetic energy cutoff was set to a large 700 eV. Electronic exchange and correlation were described using the PBEsol functional.^99^ To also include van-der-Waals interactions, D3 correction with Becke–Johnson damping^100,101^ was applied. The **k**-point meshes were generated using the Monkhorst–Pack scheme^102^ which yielded a 9 × 11 × 9 mesh for H_2_CO_3_, 17 × 7 × 7 for NaHCO_3_ and 7 × 11 × 9 for Na_2_CO_3_. Brillouin-zone integrations were performed using Blöchl's tetrahedron method.^103^ The Local Orbital Basis Suite Towards Electronic-Structure Reconstruction (LOBSTER) 5.1.1^9–11,49,52,104^ package was then used to project the PAW wave function onto the local, all-electron, contracted multiple-*ζ* Slater-type orbital basis set pbeVASPfit2015^11^ to gain the chemical information provided in this article. The basis set itself rests on prior many-body work^105^ known for supreme accuracy in terms of energy (μHartrees) and density (0.002% average error).^106^ Local orbitals were selected to correspond to the minimal basis of the respective pseudopotentials, a fundamental choice allowing for self-consistency in the end. The calculation of Bader charges was performed with the program Critic2 1.2^74,75^ using Henkelman integration.^107–109^ For technical reasons, the Bader-DI were calculated using Critic2 interfaced with Quantum ESPRESSO^60–62^ as this method is not supported for VASP. All other output was ensured to be consistent with both DFT programs.

## Author contributions

Peter C. Müller: conceptualization, investigation, writing – original draft, writing – review & editing, project administration. Linda S. Reitz: conceptualization, investigation, writing – original draft, writing – review & editing. David Hemker: conceptualization, writing – original draft. Richard Dronskowski: resources, writing – review & editing, supervision, funding acquisition.

## Conflicts of interest

There are no conflicts to declare.

## Data Availability

The original data supporting this perspective article have been generated (and may be reproduced) from the LOBSTER code which can be downloaded freely at http://www.cohp.de, based on the crystallographic data for carbonic acid, sodium hydrogen carbonate, and sodium carbonate published in ref. 69–71.
